# Dynamic Tensile Behavior of Steel HRB500E Reinforcing Bar at Low, Medium, and High Strain Rates

**DOI:** 10.3390/ma13010185

**Published:** 2020-01-02

**Authors:** Xiang Zeng, Jingsi Huo, Haitao Wang, Zhan Wang, Mohamed Elchalakani

**Affiliations:** 1 College of Civil Engineering and Architecture, Hainan University, Haikou 570228, China; 2 College of Civil Engineering, Huaqiao University, Xiamen 361021, China; huojingsi@hqu.edu.cn; 3 College of Civil Engineering, Hunan University, Changsha 410082, China; 4 The Department of Civil, Environmental and Mining Engineering, Faculty of Engineering, Computing and Mathematics, The University of Western Australia, Perth 6009, Australia

**Keywords:** strain rate, reinforcing bar, HRB500E, dynamic tensile test, constitutive model

## Abstract

The strain rate effect of engineering materials should be considered in the assessment of the performance of reinforced concrete (RC) structures under extreme dynamic loads such as blast and impact. However, the strain rate behavior of 500 MPa-grade anti-earthquake hot-rolled high-strength ribbed bar (HRB500E), used in critical RC members to improve the anti-earthquake performance, has not been investigated and reported in the open literature. That restricts its application in RC structures subjected to extreme dynamic loads. In this paper, dynamic tensile tests of HRB500E steel were conducted using an electromechanical universal testing machine and a servo-hydraulic high-speed testing machine. The stress–strain curves at strain rates ranging from 0.00025 to 550 s^−1^ were obtained where HRB500E steel was found significantly sensitive to strain rate. Existing formulations to evaluate the dynamic increase factor for yield stress (DIF_y_) are found to be not suitable for HRB500E steel, thus the widely used Cowper–Symonds and Malvar models for predicting the DIF_y_ were modified based on the test results. Furthermore, the parameter of the Mander material model for describing engineering stress–strain relationship was also calibrated. Finally, the Johnson-Cook and proposed constitutive models for the true stress–strain relationship were examined. The proposed constitutive model can provide better prediction accuracy for yield stress than the Johnson-Cook model.

## 1. Introduction

Recently, the application of high-strength reinforcing bars to reinforced concrete (RC) structures has been increasing gradually. Using high-strength reinforcing bars provides lots of benefits such as reducing steel consumption, saving natural resources, energy, and protecting the environment, as well as reducing the volumetric ratio of transverse reinforcement in RC joints to conveniently cast and compact concrete. Furthermore, the anti-earthquake reinforcing bars with high ductility, i.e., having higher ratio of ultimate tensile strength (*f*_t_) to yield stress (*f*_y_) and larger total elongation at ultimate tensile strength, have been developed [1,2,3,4,5] and employed to improve the earthquake-resistant performance of RC structures. Therefore, the anti-earthquake high-strength reinforcing bar has become a vital construction material for RC structures. In order to promote its application in RC structures and provide the basis for evaluating the structural resistance, comprehensive research on the mechanical behavior is needed, including the strain rate behavior of anti-earthquake high-strength reinforcing bar subjected to high dynamic loads.

It has been proved experimentally that steel is significantly sensitive to the strain rate induced by dynamic loads [6,7,8]. Furthermore, it is possible for engineering structures to be exposed to extreme dynamic loads during their service life, such as vehicle or vessel collision, gas or bomb explosion [9], and progressive collapse [10,11,12]. Therefore, understanding the strain rate behavior and obtaining the rate-dependent constitutive model of reinforcing bar are of significance for the design and performance evaluation of RC structures under such extreme dynamic loads. Even in the case of seismic loading conditions with relative low strain rate in materials, the strain rate effect on the performance of RC structures is significant to be considered, which was discussed in some literature [13,14]. The HRB500E reinforcing bar, namely 500 MPa-grade anti-earthquake hot-rolled high-strength ribbed bar, meeting the requirements described in Table 1, has been introduced into voluntary Chinese standard GB/T 1499.2-2018 [1], and is being popularized for improving the anti-earthquake performance of RC structures and saving the natural resources. However, its important strain rate behavior has not been experimentally studied, which makes it difficult to evaluate the dynamic performance of RC structures subjected to the extreme dynamic loads.

**Figure 1:**
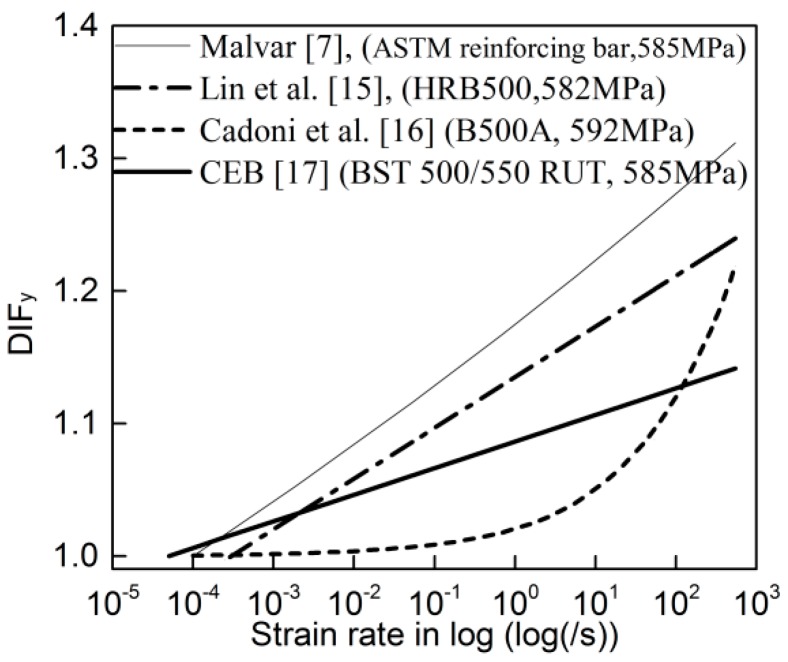
The dynamic increase factor for yield stress (DIF_y_) predicted by different formulations for 500 MPa-grade reinforcing bars.

The strain rate behavior of 500 MPa-grade reinforcing bars from different manufacturers from different countries has been studied, and various formulations to evaluate the dynamic increase factor for yield stress (DIF_y_) were established based on the test results [7,15,16,17]. Even though the formulations were established based on the test data of reinforcing bars with the same strength grade, they were quite different when they were utilized to predict the DIF_y_ with yield stress of 585 MPa, as shown in Figure 1. Here, the reason for adopting the yield stress of 585 MPa is that the yield stress for 500 MPa-grade reinforcing bars in literature [7,15,16,17] was about between 582 and 592 MPa, and 585 MPa is about the average of them. These wide variations in DIF_y_ are most likely due to the different chemical composition and microstructure. To this end, it is dubious to assess the strain rate behavior of HRB500E reinforcing bar based on the existing research results, and it is essential to conduct a new experimental study to understand the dynamic behavior.

The aim of the present study was to investigate the dynamic tensile behavior of HRB500E steel and to obtain the rate-dependent constitutive model. An electromechanical universal testing machine and a servo-hydraulic high-speed testing machine were used to conduct dynamic tensile tests on HRB500E reinforcing bars. The dynamic tension tests covered the strain rates ranging from 0.00025 to 550 s^−1^. Based on the test results, the models of dynamic increase factors for yield stress and ultimate tensile strength, as well as constitutive models for the engineering stress−strain relationship and true stress–strain relationship were investigated and proposed. The research results will be of practical value for analyzing and investigating the dynamic performance of RC structures subjected to dynamic loads.

## 2. Experimental Program

### 2.1. Specimen Details

The material used in this test is HRB500E reinforcing bar produced by Jiangsu Yonggang Group Co., Ltd. in Suzhou, China. The chemical composition and carbon equivalent value (CEV) of the test material are presented in Table 2, meeting the requirements of GB/T 1499.2-2018 [1].

**Figure 2:**
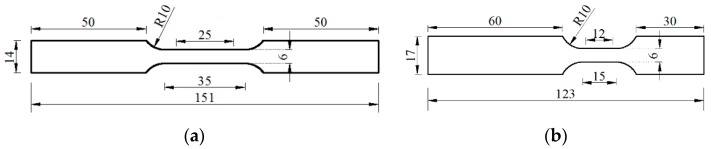
Specimen geometry (unit: mm): (**a**) quasi-static tensile specimen; (**b**) dynamic tensile specimen.

The sheet-type specimen was adopted in order to fulfill the standard ISO (International Organization for Standardization) 26203-2:2011 [18] for high-strain-rate tensile test using servo-hydraulic test systems. Thin sheets with 3 mm thickness were firstly sampled symmetrically from the center of sections of reinforcing bars. Then test specimens were further fabricated using the thin sheets. The geometry of specimens for quasi-static tensile test and dynamic tensile test, which were determined according to American Society of Testing Materials (ASTM) E8/E8M-16a [19] and ISO 26203-2:2011 [18] respectively, are shown in Figure 2. Moreover, the gauge lengths for quasi-static and dynamic tensile specimens are 25 and 12 mm, respectively.

### 2.2. Test Arrangement

Totally, twenty four effective specimens at eight strain rates were tested under monotonic load though the tensile testing machines. At least three specimens for a group were tested at each strain rate. Where the consistency of the three tests is not proven to be satisfactory (a deviation of more than 5% from the average value of yield stress of the three samples), more tests are carried out till data consistency is achieved.

An electromechanical universal testing machine with a load capacity of 50 kN was used to perform the quasi-static and relatively low-strain-rate tensile tests with the strain rates lower than 0.1 s^−1^. Quasi-static mechanical properties were obtained through the standard uniaxial tensile tests under the strain rate of 0.00025 s^−1^ following ASTM E8/E8M-16a [19]. Meanwhile, the Vic-3D non-contact full-field strain measurement system by Correlated Solutions, Inc. (Irmo, SC, USA) was utilized to acquire the mean strain of the gauge length. For the in-plane strain measurement in the test, the measurement system contains a camera with a resolution of 2048 × 1088 and a frame rate of 100 fps, a Vic-Snap image acquisition software to allow for data acquisition and external data synchronization and a VIC-2D digital image correlation software (Version 2009.1.0, Correlated Solutions Inc., Irmo, SC, USA) for processing the acquired images to obtain the strain. Prior to the test, a random artificial speckle pattern was created in the parallel length of the specimen using dotted-effect black spray paint.

**Figure 3:**
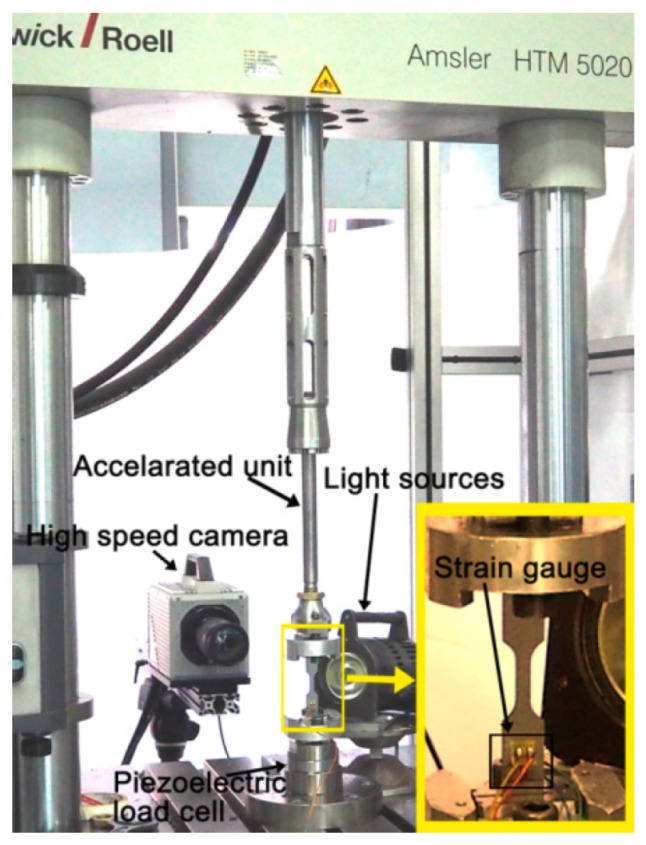
Tension test setup for medium and high strain rate.

A Zwick/Roell HTM5020 servo-hydraulic high-speed testing machine (Ulm, Germany), (Figure 3) was utilized to implement the medium- and high-strain-rate tensile tests with the strain rate ranging from 0.1 to 550 s^−1^. The high-speed tensile testing machine has a load capability of 50 kN and a maximum loading velocity of 20 m/s. A piezoelectric-type load cell with a capacity of 200 kN was used to measure the load when the strain rate is lower than 100 s^−1^. For higher strain rates, two strain gauges (Figure 3) attached on both sides of the longer grip end of the specimen were used to measure the tensile force [20] to make up the shortcoming that the natural frequency of piezoelectric load cell cannot achieve the accurate force measurement [18]. The foil strain gauge with an electric resistance of 120 ohms and a gauge factor of 2.1 was adopted in tests. The Vic-3D non-contact full-field strain measurement system combined with the FASTCAM SA1.1 high-speed video camera (San Diego, CA, USA), which can capture images at a maximum frame rate of 675,000 fps and a submicrosecond exposure to eliminate motion blur, was used to acquire the engineering strain of gauge length. The maximum data acquisition rate in the tests was 125,000 Hz.

## 3. Results

**Figure 4:**
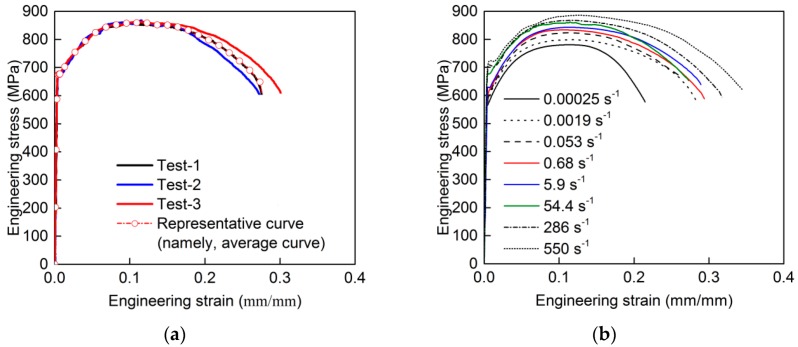
Engineering stress–strain curves of the HRB500E reinforcing bar: (**a**) Representative engineering stress–strain curve at a strain rate of 54.4 s^−1^; (**b**) representative engineering stress–strain curves at different strain rates.

As demonstrated in Figure 4a, the test results have high reproducibility. For each group of specimens, the mean value of measured strain rates of repeated specimens was taken as the representative strain rate. The representative engineering stress–strain curve for each group of specimens at different strain rates was determined by averaging the engineering stress–strain curves of repeated specimens, as illustrated in Figure 4a. All the representative engineering stress–strain curves of the HRB500E reinforcing bar at different strain rates are shown in Figure 4b, and it can be seen that HRB500E steel is significantly sensitive to the strain rate.

**Figure 5:**
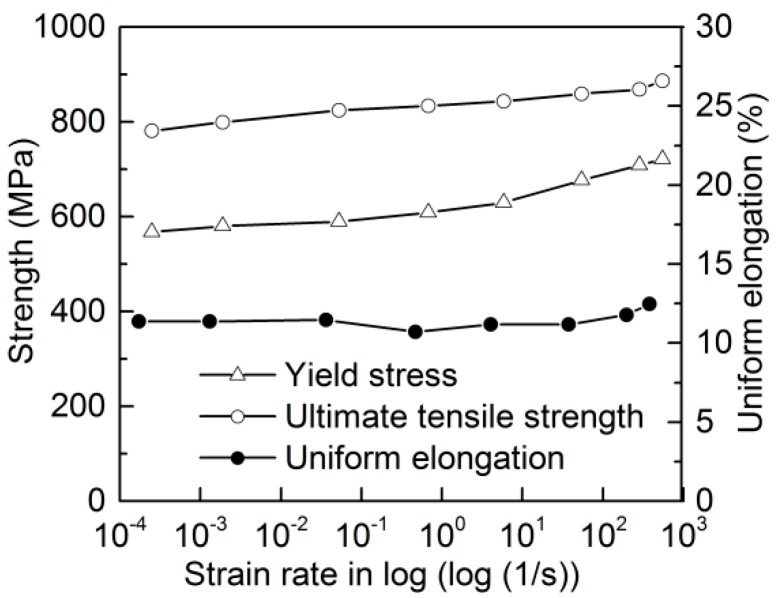
Variations of yield stress, ultimate tensile strength, and uniform elongation with increasing strain rate in log scale.

Yield stress is described by the proof stress corresponding to 0.2% plastic strain. The yield stress (*f*_y_), ultimate tensile stength (*f*_t_), and uniform elongation (namely the total elongation at ultimate tensile strength, *ε*_u_) are summarized in Table 3. The maximum deviation from the average yield stress and ultimate tensile strength for all the test groups was no more than 4%. In Figure 5, the yield stress, ultimate tensile strength, and uniform elongation are plotted as a function of strain rate. The figure shows that the yield stress increases nonlinearly with the increasing logarithmic strain rate, while the ultimate tensile strength has a notable linear relationship with the increasing logarithmic strain rate. As the strain rate increases, there is no monotonical change in uniform elongation. Yield stress and ultimate tensile strength increased apparently, from 567 to 721 MPa (increased by 27.2%) and from 781 to 886 MPa (increased by 13.4%), respectively, when the strain rate increased from 0.00025 to 550 s^−1^.

## 4. Dynamic Increase Factors (DIFs) for Yield Stress and Ultimate Tensile Strength

### 4.1. Comparison of DIFs among Test Results on 500 MPa-Grade Reinforcing Bars

**Figure 6:**
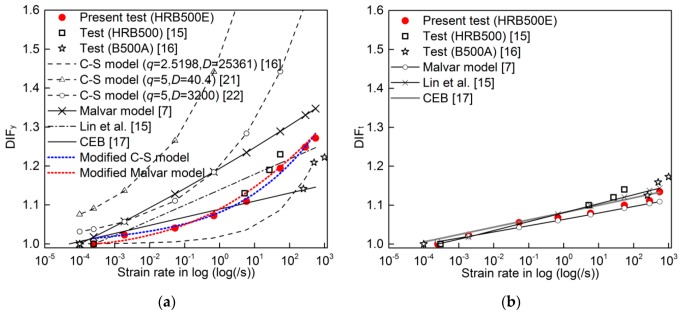
Comparison of DIFs from models and test results: (**a**) DIF_y_; (**b**) DIF_t_.

DIFs for yield stress and ultimate tensile strength versus strain rate curves of HRB500E steel are depicted in Figure 6. Meanwhile, the existing test results of 500 MPa-grade hot-rolled ribbed bar (HRB500) [15] and cold-worked reinforcing bar (B500A) [16] are also shown in Figure 6 for comparison. The figure indicates that the DIF_y_ is larger than DIF_t_ (DIF for ultimate tensile strength). That means that the yield stress is more sensitive to strain rate than the ultimate tensile strength.

In Figure 6a, it is seen that DIF_y_ of B500A steel is lower than the DIF_y_ of HRB500E and HRB500 steel in the range of test strain rates. While B500A steel bar is cold-worked steel from hot-rolled wire rod and available as coils or bars with lower strength than 500 MPa, HRB500E and HRB500 steel bars are hot rolled. Cold work leads to microstructural changes in the crystal lattice, including increased dislocation density and changed grain size distributions, which results in an increase in yield strength and a decrease in ductility. Therefore, the different chemical compositions and microstructures are mainly responsible for the discrepancy of the dynamic behaviors among them.

Although it is observed that the DIF_y_ of HRB500E steel is very close to the DIF_y_ of HRB500 steel in the limited range of test strain rates, the trends of DIF_y_ between HRB500E and HRB500 with increasing strain rate seem to be different over a wider strain rate range. From the aspect of quasi-static behavior, it has been known that the HRB500E reinforcing bar developed for improving anti-earthquake performance of RC structures has higher ductility than HRB500 reinforcing bar [1]. In addition, the chemical compositions and microstructures are distinct between them. Due to the lack of sufficient dynamic tensile test data of HRB500 steel, it remains pending whether the DIF_y_ of HRB500E steel is close to the DIF_y_ of HRB500 steel over a wide strain rate range.

Figure 6b shows that the difference in DIF_t_ among HRB500E, HRB500, and B500A steel is limited in the test strain rate range. The maximum difference of DIF_t_ between them is less than 5%. However, it is observed that the trends of DIF_t_ with strain rate increase are different among them.

### 4.2. Comparison of DIFs between Test Results and Existing Models

Several existing models [7,15,16,17,21,22] are employed to predict the DIF_y_ and DIF_t_ of HRB500E steel. The Cowper-Symonds (C-S) model [21], a common and widely used model for DIF_y_, is described as:

(1) $${\mathrm{DIF}}_{y}=\frac{f_{\mathrm{dy}}}{f_{\mathrm{sy}}}=1+{\left(\frac{\dot{\varepsilon }}{D}\right)}^{\frac{1}{q}},$$

where *f*_sy_ and *f*_dy_ are quasi-static and dynamic yield stress, respectively, and $\dot{\varepsilon }$ is strain rate. For mild steel, material coefficients *q* and *D* are 5 and 40.4, respectively [21]. For high tensile steel, *D* and *q* are taken as 3200 and 5, respectively [22]. For B500A steel, three groups of *D* and *q* were obtained, which are corresponding to the three different types of rebars (*d* = 6, 8, 10 mm) tested by Cadoni et al. [16]. It is found that the DIF_y_ predicted by C-S models with the three groups of *D* and *q* are close to each other, and the DIF_y_ from *D* and *q* for *d* = 8 mm is almost the average value of the DIF_y_ from the three groups of *D* and *q*. Thus, *D* = 25,361 and *q* = 2.519 for B500A steel bar with a diameter of 8 mm [16] were adopted to calculate the DIF_y_ of HRB500E steel, as shown in Figure 6a.

Based on the dynamic tensile test results of reinforcing bars with quasi-static yield stress between 290 and 710 MPa, Malvar [7] proposed the following formulations for DIF_y_ and DIF_t_:

(2) $$\mathrm{DIF}={\left(\frac{\dot{\varepsilon }}{10^{-4}}\right)}^{\alpha },$$

where for yield stress, *α* = *α*_y_:

(3) $$\alpha_{y}=0.0074-0.040\frac{f_{\mathrm{sy}}}{414},$$

and for ultimate tensile strength, *α* = *α*_t_:

(4) $$\alpha_{t}=0.019-0.009\frac{f_{\mathrm{sy}}}{414}.$$

In Equation (2), the denominator 10^−4^ means the quasi-static strain rate with the unit s^−1^. The test strain rate ranges from 10^−4^ to 10 s^−1^ for formulations (2)–(4). Moreover, the formulations (2)–(4) have been introduced into Model Code 2010 [9] and are applied to a wider strain rate range of 10^−4^ to 10^3^ s^−1^, whereas there is no sufficient test data supporting the application at a strain rate lager than 10 s^−1^.

The Comité Euro-International du Béton (CEB) Bulletin 187 [17] provided the formulations for DIF_y_ and DIF_t_ with the general form:

(5) $$\mathrm{DIF}=1+\frac{m}{f}\ln\left(\frac{\dot{\varepsilon }}{{\dot{\varepsilon }}_{0}}\right),$$

where ${\dot{\varepsilon }}_{0}$ is quasi-static strain rate (${\dot{\varepsilon }}_{0}$ = 0.00001 s^−1^), *f* = *f*_sy_ for DIF_y_, and *f* = *f*_st_ for DIF_t_. The parameter *m* has different values for different reinforcing bars. For the hot-rolled reinforcing bar with 500 MPa grade, *m* = *m*_y_ = 5.1 for DIF_y_ and *m* = *m*_t_ = 6.4 for DIF_t_. The formulations and parameter were acquired on the basis of the test results of reinforcing bars under strain rates lower than 10 s^−1^. In Reference [15], ${\dot{\varepsilon }}_{0}$ is taken as 0.0003 s^−1^, *m*_y_ and *m*_t_ were calibrated to be 9.72 and 7.78 for HRB500 steel, respectively, based on the tensile test results of HRB500 at intermediate strain rates between 5.2 and 54.2 s^−1^.

The comparison of DIF_y_ and DIF_t_ between the test results and the aforementioned models [7,15,16,17,21,22] is presented in Figure 6. In Figure 6a, it is shown that the C-S model in [16,21,22] overestimates or underestimates the DIF_y_ due to the inappropriate values of *q* and *D* for HRB500E. However, its nonlinear characteristic of DIF_y_ with increasing logarithmic strain rate is similar with that of HRB500E steel. The C-S model is needed to be calibrated for precisely predicting the DIF_y_ of HRB500E.

In a limited strain rate range such as from 10^−4^ to 10 s^−1^ or from 10 to 550 s^−1^, approximate linear relationships between DIF_y_ and logarithmic strain rate for HRB500E are observed in Figure 6a. However, the relationship is evidently nonlinear over the wider range from low to high strain rate. Equations (2)–(5) [7,15,17] proposed on the basis of the test results in limited-strain-rate ranges describe the linear relationships, which are against the nonlinear tendency of DIF_y_ with increasing logarithmic strain rate. Therefore, the formulations are unsuitable for predicting the DIF_y_ of HRB500E in large strain rate range, as shown in Figure 6a. Moreover, it is worth further discussing whether it is rational for Equations (2)–(4) to be applied to the strain rate range larger than 10 s^−1^ by Mode Code 2010 [9] in the absence of sufficient test data over a wide-strain-rate range.

As shown in Figure 6b, Equations (2)–(4) [7] and Equation (5) [15,17] seem to be suitable for predicting DIF_t_ for HRB500E. A comparison of DIF_t_ predicted by the existing models was shown in Table 4, which indicates that the predicted results from the different models are in good agreement with test results.

### 4.3. Calibration of Models for DIF_y_

For better predicting the dynamic yield stress, the widely used C-S model and Malvar model were calibrated. New values of material coefficients *q* and *D* in C-S model and *m* in Malvar model can be obtained by fitting the models with test data.

**Figure 7:**
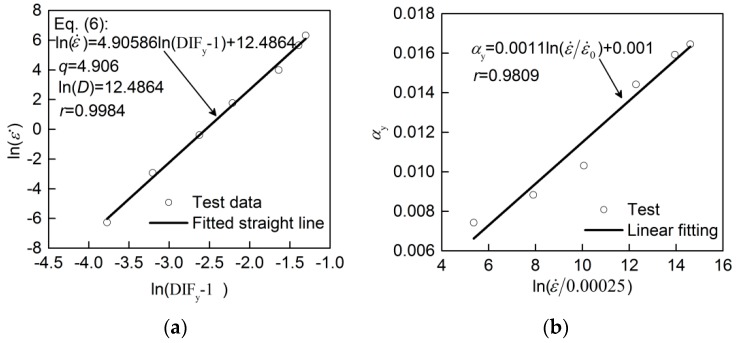
Fitting parameters of models for DIF_y_: (**a**) material coefficients, *D* and *q,* in C-S model; (**b**) *α*_y_ in Malvar model.

In order to fit the C-S model conveniently, Equation (1) was transformed into Equation (6). The relationship between ln($\dot{\varepsilon }$) and ln(DIF_y_-1) is demonstrated in Figure 7a. Through the linear fitting method, *D* = 264,713 and *q* = 4.906 were determined. Figure 6a illustrates that the modified C-S model (listed in Table 5) has high prediction accuracy for DIF_y_ with the largest error of 1.5%.

(6) $$\ln\left(\dot{\varepsilon }\right)=q\ln\left({\mathrm{DIF}}_{y}-1\right)+\ln\left(D\right)$$

For the Malvar model (Equation (2)), the denominator 10^−4^ s^−1^ in Equation (2), representing the quasi-static strain rate ${\dot{\varepsilon }}_{0}$, is substituted by ${\dot{\varepsilon }}_{0}$ = 0.00025 s^−1^ in this study. Transforming Equation (2) into Equation (7), it was found that the relationship between ln($\dot{\varepsilon }$) and ln(DIF_y_) is nonlinear according to the test results, which indicates that parameter *α*_y_ is non-constant. From Equation (7), *α*_y_ can be expressed by Equation (8). Based on the test data, *α*_y_ was calculated by using Equation (8), and then was depicted as the increasing $\dot{\varepsilon }/0.00025$ in Figure 7b. Through linearly fitting the relation of *α*_y_ to $\ln(\dot{\varepsilon }/0.00025)$ as shown in Figure 7b, the expression of *α*_y_ (Equation (9)) was acquired, where *α*_y_ is quite different from the constant in the previous model. As shown in Figure 6a, the values for DIF_y_ predicted by the modified Malvar model with Equation (9) (listed in Table 5) have a good agreement with the test DIF_y_ with the largest error of 1.8%.

(7) $$\ln\left(\dot{\varepsilon }\right)=\frac{1}{\alpha_{y}}\ln\left({\mathrm{DIF}}_{y}\right)+\ln\left(0.00025\right)$$

(8) $$\alpha_{y}=\frac{\ln\left({\mathrm{DIF}}_{y}\right)}{\ln\left(\dot{\varepsilon }/0.00025\right)}$$

(9) $$\alpha_{y}=0.0011\ln(\dot{\varepsilon }/0.00025)+0.001$$

## 5. Rate-Dependent Constitutive Model

### 5.1. Constitutive Model for Engineering Strain Hardening Curve

**Figure 8:**
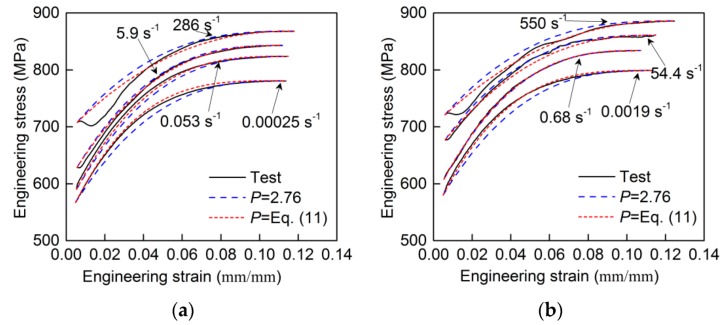
Comparison between test curves and modified Mander model: (**a**) 0.00025, 0.053, 5.9 and 286 s^−1^; (**b**) 0.0019, 0.68, 54.4 and 550 s^−1^.

The engineering strain hardening curves from tests are shown in Figure 8. In this paper, the Mander model, as described by Equation (10), was used as the constitutive model for the engineering strain hardening curve. Mander [23] first proposed the quasi-static form of Equation (10) for describing the strain hardening curve of mild steel bar. Then, the application was extended to the dynamic loading conditions [24].

(10) $$\sigma =f_{\mathrm{dt}}-(f_{\mathrm{dt}}-f_{\mathrm{dy}}){\left(\frac{\varepsilon_{\mathrm{du}}-\varepsilon }{\varepsilon_{\mathrm{du}}-\varepsilon_{\mathrm{dh}}}\right)}^{P}\;for\;\varepsilon_{\mathrm{dh}}<\varepsilon \leq \varepsilon_{\mathrm{du}},$$

where parametric *P* is the strain hardening power, *ε*_dh_ and *ε*_du_ are the strain at the 0.2% proof stress and uniform elongation, respectively.

**Figure 9:**
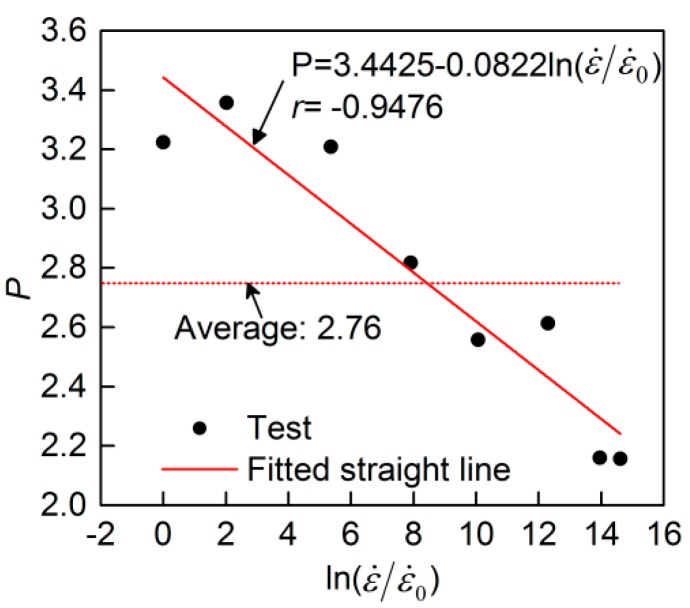
Relationship of parameter *P* to $\ln(\dot{\varepsilon }/{\dot{\varepsilon }}_{0})$

To determine the strain hardening power, *P*, in Equation (10), the value of *P* for strain hardening curve at each strain rate was firstly obtained by fitting the curves with Equation (10). From Figure 9, it is seen that parameter, *P*, at various strain rates is not a constant, but ranges between 2.1 and 3.4. Generally, parameter *P* is taken as a constant for describing the stress–strain curves at various strain rates, which can simplify Equation (10). In that case, the mean value of parameter *P* at various strain rates is adopted, which is 2.76 here. However, taking parameter *P* as a constant in Equation (10) leads to a deviation from its value at different strain rates, and thus decreases the prediction accuracy. Therefore, a more precise description of parameter *P* was proposed, adopting Equation (11) between *P* and $\ln(\dot{\varepsilon }/{\dot{\varepsilon }}_{0})$. Equation (11) was obtained by linear fitting, as shown Figure 9. Figure 8 shows the comparison between test curves and the modified Mander model with different *P*. It can be seen that the curves derived from the model with a constant *P* = 2.67 have acceptable accuracy. Taking *P* as a constant makes the expression of the Mander model simple, but taking *P* as a variable expressed as Equation (11) makes the model have a better agreement with the test results.

(11) $$P=3.4425-0.0822\ln(\dot{\varepsilon }/{\dot{\varepsilon }}_{0})$$

### 5.2. Constitutive Model for the True Stress–Strain Relationship

#### 5.2.1. Johnson-Cook (J-C) Model

The formulation of the J-C model [25] is described as

(12) $$\sigma =[A+B{\left(\varepsilon_{p}\right)}^{n}][1+C\ln({\dot{\varepsilon }}^{*})][1-T^{*}{}^{m}],$$

where *ε*_p_ is the equivalent plastic strain, ${\dot{\varepsilon }}^{*}=\dot{\varepsilon }/{\dot{\varepsilon }}_{0}$ (quasi-static strain rate ${\dot{\varepsilon }}_{0}$ = 0.00025 s^−1^) is the dimensionless plastic strain rate, and T* is the homologous temperature. Coefficients *A*, *B*, *C*, *n*, *m* are the material constants. The third bracket represents the temperature effect, which is taken as 1 since temperature effects are not considered in this study.

The first bracket represents the quasi-static flow stress *σ*_s_ (*σ*_s_ = A + B (*ε*_p_)*^n^*), and material constants *A*, *B*, *n* can be determined by fitting the quasi-static stress–strain curves. *A* is the quasi-static yield stress, and *B* and *n* describe the response of strain hardening.

The second bracket represents the strain rate effect, which is the dynamic increase factor of quasi-static flow stress. Here, coefficient *C* is usually determined by fitting Equation (13) or (14) with test data. In the standard J-C model, *C* is regarded as a constant. However, the relationships described by Equations (13) and (14) are nonlinear in some cases, and thus *C* is modified as a variable related to the strain rate [26,27]. Whether *C* is a variable is dependent on the behavior of steel material.

(13) $$\sigma /\sigma_{s}=1+C\ln({\dot{\varepsilon }}^{*})$$

(14) $$\bar{\sigma /\sigma_{s}}=1+C\ln({\dot{\varepsilon }}^{*})$$

**Figure 10:**
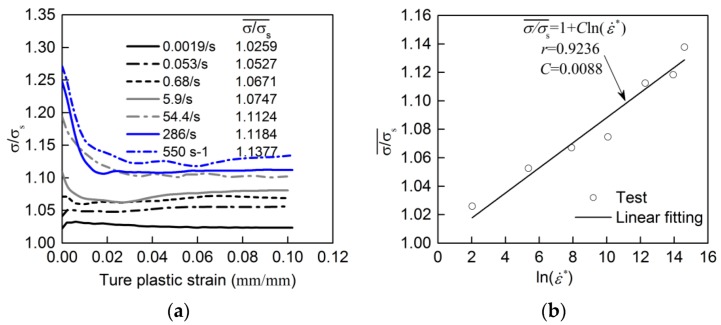
Determining coefficient *C* for Johnson-Cook (J-C) model: (**a**) *σ*/*σ*_s_ at various strain rates; (**b**) determining coefficient *C*.

There exist two methods to determine *C* [26,27,28,29], which correspond to Equations (13) and (14) respectively:

(1) σ and σ_s_ are taken as the dynamic and quasi-static yield stress respectively [27,28], which are corresponding to zero plastic strain. Thus, σ/σ_s_ represents the DIF_y_. Based on the test DIF_y_ and $\ln({\dot{\varepsilon }}^{*})$, *C* can be calculated through Equation (13). This method to determine *C* was not preferred in this study because DIF_y_ markedly overestimates the increase in ultimate tensile strength (indicated by Figure 6, DIF_y_ is much larger than DIF_t_).
(2) σ and *σ*_s_ are taken as the dynamic and quasi-static flow stress related to plastic strain, respectively [26,29]. The ratio σ/σ_s_ at the same true plastic strain was firstly calculated. In Figure 10a, *σ*/*σ*_s_ versus true plastic strain curves at various strain rates were described. Then, the mean value of *σ*/*σ*_s_ over the entire range of true plastic strain at each strain rate, denoted as $\bar{\sigma /\sigma_{s}}$ (in Equation (14)), was calculated and given in Figure 10a. In Figure 10b, the relationship between $\bar{\sigma /\sigma_{s}}$ and $\ln({\dot{\varepsilon }}^{*})$ was depicted in approximate linearity. By linearly fitting Equation (14) with the data in Figure 10b, *C* was determined to be 0.0088.

All parameters of the J-C model for HRB500E steel are listed in Table 6.

#### 5.2.2. Proposed Constitutive Model

The proposed constitutive model is expressed as

(15) $$\sigma =A\cdot {\mathrm{DIF}}_{y}+B{\left(\varepsilon_{p}\right)}^{n}\cdot \bar{f({\dot{\varepsilon }}^{*})},$$

where parameters *A*, *B*, and *n* represent the same meanings with those in Equation (12). At a certain strain rate, the flow stress at any plastic strain *ε*_p_ is the summation of yield stress (*A*∙DIF_y_) and plastic part of the stress ($B{\left(\varepsilon_{p}\right)}^{n}\cdot \bar{f({\dot{\varepsilon }}^{*})}$). DIF_y_ can be calculated using the C-S model or the Malvar model (described in Table 5). $\bar{f({\dot{\varepsilon }}^{*})}$ is a function of strain rate and represents the average effect of strain rate on strain hardening curve at a strain rate of $\dot{\varepsilon }$. $\bar{f({\dot{\varepsilon }}^{*})}$ is determined by the average of $f({\dot{\varepsilon }}^{*})$, where $f({\dot{\varepsilon }}^{*})$ denotes the ratio of dynamic flow stress to quasi-static flow stress (the stress excluding *A*∙DIF_y_) at the same plastic strain *ε*_p_ under the strain rate of $\dot{\varepsilon }$. $f({\dot{\varepsilon }}^{*})$ can be calculated by Equation (16).

(16) $$f({\dot{\varepsilon }}^{*})=\frac{\sigma -A\cdot {\mathrm{DIF}}_{y}}{B{\left(\varepsilon_{p}\right)}^{n}}$$

**Figure 11:**
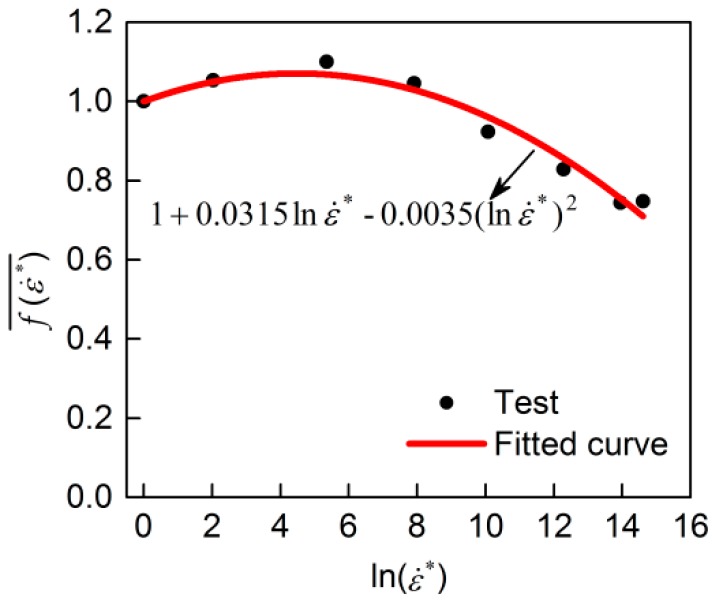
Fitting the function of $\bar{f({\dot{\varepsilon }}^{*})}$.

Under quasi-static loading, DIF_y_ and $\bar{f({\dot{\varepsilon }}^{*})}$ are equal to 1 in Equation (15), and then Equation (15) becomes $\sigma =A+B{\left(\varepsilon_{p}\right)}^{n}$, which describes the quasi–static curve. Thus, *A*, *B*, and *n* were firstly obtained by fitting the quasi–static curve (given in Table 7). After obtaining *A*, *B*, and *n*, $f({\dot{\varepsilon }}^{*})$ was calculated by Equation (16) at any plastic strain *ε*_p_ at a strain rate of $\dot{\varepsilon }$, and then $\bar{f({\dot{\varepsilon }}^{*})}$ for each strain rate was calculated. Through fitting the relationship between $\bar{f({\dot{\varepsilon }}^{*})}$ and $\ln({\dot{\varepsilon }}^{*})$ as shown in Figure 11, the expression of $\bar{f({\dot{\varepsilon }}^{*})}$ was acquired as shown in Table 7.

**Figure 12:**
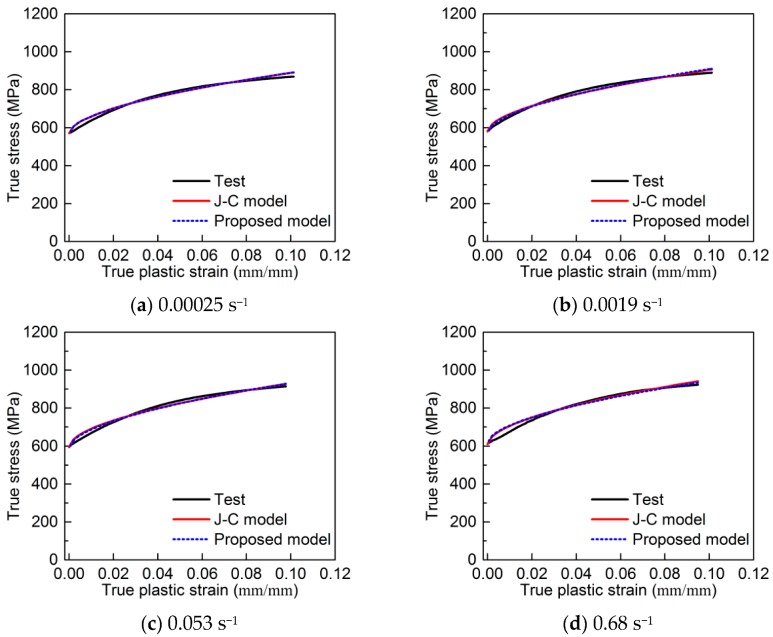

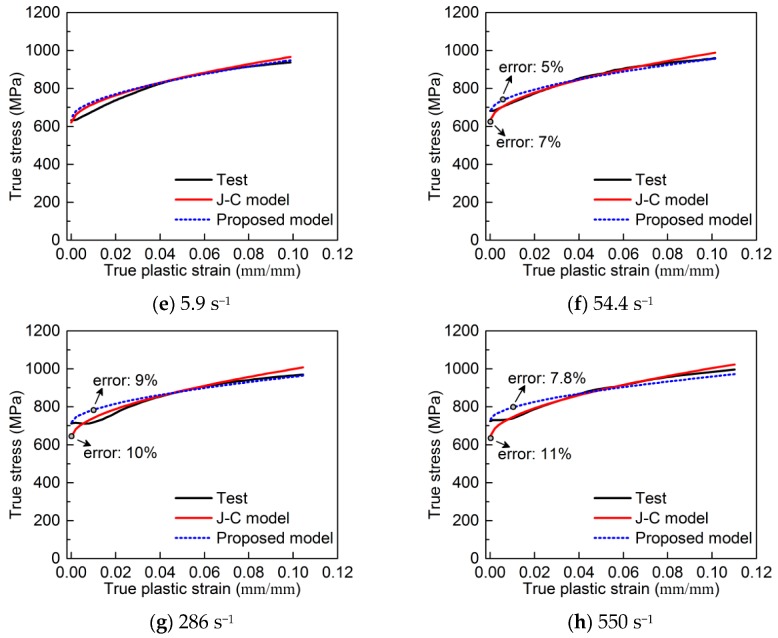
Comparison between test results and predicted true stress–plastic strain curves: (**a**) 0.00025 s^−1^; (**b**) 0.0019 s^−1^; (**c**) 0.053 s^−1^; (**d**) 0.68 s^−1^; (**e**) 5.9 s^−1^; (**f**) 54.4 s^−1^; (**g**) 286 s^−1^; (**h**) 550 s^−1^.

A comparison among the J-C model, the proposed constitutive model and the test true stress–plastic strain curves is shown in Figure 12. It is seen that the two constitutive models are in good agreement with the test results. The predicted errors for the two models are limited, which is shown in Figure 12. However, the two models have different features. The curves predicted by the two models almost overlap with each other at strain rates between 0.00025 and 5.9 s^−1^ (Figure 12a–e). As the strain rate increases from 54.4 to 550 s^−1^, the discrepancy between the two models gradually increases. The J-C model predicts the stresses well in the middle sections of the curves, but it underestimates and overestimates the stresses in the initial sections and end sections of the curves, respectively. The maximum error for the J-C model occurs at the yield point and increases with the strain rate increase (Figure 12f–h). It reaches to the maximum value of 11% at 550 s^−1^. In order to predict the yield stress more accurately, the new model was proposed. From Figure 12, it is seen that the proposed model provides high prediction accuracy for yield stress. It initially over-predicts the stresses up to about a plastic strain of 0.04 and then it matches the test curves well. The error of over-predicted stresses from the proposed model is below 9% for all curves.

## 6. Conclusions

A series of dynamic tensile tests on HRB500E reinforcing bar were conducted over a wide strain rate range (0.00025–550 s^−1^). The test results show that HRB500E reinforcing bar is significantly sensitive to strain rate, and the yield stress and ultimate tensile strength increase by 27.2% and 13.4% respectively from 0.00025 to 550 s^−1^. Based on the test results, the following conclusions can be drawn:

- The linear relationship between dynamic increase factor for yield stress (DIF_y_) and logarithm of strain rate is observed in a limited strain rate range (from 10^−4^ to 10 s^−1^ or from 10 to 550 s^−1^). However, the relationship is markedly nonlinear from low to high strain rates. Thus, incorrect DIF_y_ may be obtained if the application range of the DIF_y_ models, acquired based on the test results in a limited strain rate range, is extended to a wider strain rate range. It is essential to conduct the dynamic tests over a wide-strain-rate range in order to comprehensively understand the rate-dependent behavior of steel.
- Existing models of DIF_y_ are examined to be inconsistent with the test results of HRB500E in the wide range of test strain rate. By contrast, the existing models of dynamic increase factor for tensile strength (DIF_t_) described in the aforementioned literature are in good agreement with the test results. The commonly used Cowper-Symonds and Malvar models for DIF_y_ were accurately modified and demonstrated to provide excellent predictions. Different from the existing Malvar model with a constant exponent, the modified Malvar model with a variable exponent is more suitable for HRB500E reinforcing bar.
- Mander model was calibrated to predict the engineering stress–strain hardening curve and the material parameter, *P*, was fitted and evaluated. The model which takes the parameter *P* as a variable agrees with the test curves better than the model with the constant *P*.
- The parameters of Johnson-Cook and proposed constitutive models for the true stress–strain relationship were determined. The two models are in good agreement with the test results. The proposed model can provide better prediction accuracy for yield stress than the Johnson-Cook model. The two models for HRB500E steel are valid in the strain rate range from 2.5 × 10^−4^ to 550 s^−1^. But, more test results are needed to verify them at higher strain rates.

## Figures and Tables

**Table 1:** Requirements of standard GB/T 1499.2-2018 for anti-earthquake reinforcing steel.

*f*_y_ (MPa)	*f*_t_ (MPa)	*f*_t_/*f*_y_	Total Elongation (%)	*f*_y_/*f*_y0_
≥500	≥630	≥1.25	≥9	≤1.3

Note: *f*_y_ and *f*_y0_ represent measured yield stress and strength grade, respectively.

**Table 2:** CEV of the 500 MPa-grade anti-earthquake HRB500E (in weight %).

C	Si	Mn	P	S	Cr	V	Mo	Cu	Ni	CEV
0.249	0.37	1.407	0.026	0.013	0.045	0.118	0.009	0.010	0.008	0.519

**Table 3:** Material properties at different strain rates.

Strain Rate (s^−1^)	Yield Stress		Ultimate Tensile Strength		Uniform Elongation	
	*f*_sy_ or *f*_dy_ (MPa)	Increment (%)	*f*_st_ or *f*_dt_ (MPa)	Increment (%)	*ε*_su_ or *ε*_du_ (%)	Increment (%)
0.00025	567	0.0	781	0.0	11.4	0.0
0.0019	580	2.3	799	2.3	11.4	0.0
0.053	590	4.1	824	5.5	11.5	0.9
0.68	608	7.2	834	6.8	10.7	−6.1
5.9	629	10.9	843	7.9	11.2	−1.8
54.4	677	19.4	861	10.2	11.2	−1.8
286	708	24.9	868	11.1	11.8	3.5
550	721	27.2	886	13.4	12.5	9.6

Note: subscripts s and d mean the quasi-static and dynamic tension, respectively.

**Table 4:** Comparison of DIF_t_ predicted by existing models.

Strain Rate (s^−1^)	Test DIF_t_	Predicted DIF_t_					
		Malvar [7]	Error (%)	CEB [17]	Error (%)	Lin et al. [15]	Error (%)
0.0019	1.023	1.020	−0.3	1.030	0.7	1.018	−0.5
0.053	1.055	1.043	−1.1	1.057	0.2	1.052	−0.3
0.68	1.068	1.061	−0.7	1.078	0.9	1.077	0.8
5.9	1.079	1.076	−0.3	1.096	1.6	1.098	1.8
54.4	1.099	1.092	−0.6	1.114	1.4	1.121	2.0
286	1.111	1.104	−0.6	1.128	1.5	1.137	2.3
550	1.134	1.109	−2.2	1.133	−0.1	1.141	0.6

**Table 5:** Proposed models for DIF_y_ of HRB500E steel.

Model	Equation	Parameters
Cowper-Symonds (C-S) model	${\mathrm{DIF}}_{y}=1+{\left(\dot{\varepsilon }/D\right)}^{\frac{1}{q}}$	*D =* 264713, *q* = 4.906
Malvar model	$\mathrm{DIF}={\left(\dot{\varepsilon }/0.00025\right)}^{\alpha_{y}}$	$\alpha_{y}=0.0011\ln\left(\dot{\varepsilon }/0.00025\right)+0.001$

**Table 6:** Parameters of J-C model for HRB500E steel.

Model Parameter	*A* (MPa)	*B* (MPa)	*n*	*C*
Value	*f*_sy_	1133	0.5508	0.0088

**Table 7:** Parameters in the proposed constitutive model (Equation (15)).

Model Parameter	*A* (MPa)	*B* (MPa)	*n*	$\bar{f({\dot{\varepsilon }}^{*})}$
Value	*f_sy_*	1133	0.5508	$1+0.0315\ln{\dot{\varepsilon }}^{*}-0.0035{\left(\ln{\dot{\varepsilon }}^{*}\right)}^{2}$
